# Acceptability and Impact of an Educational App (iCare) for Informal Carers Looking After People at Risk of Pressure Ulceration: Mixed Methods Pilot Study

**DOI:** 10.2196/36517

**Published:** 2022-09-16

**Authors:** Eamonn McKeown, Caroline McGraw, Pru Holder, Jenny Shand, Shashivadan P Hirani

**Affiliations:** 1 School of Health & Psychological Sciences City, University of London London United Kingdom; 2 Faculty of Brain Sciences University College London London United Kingdom

**Keywords:** pressure ulcers, informal carers, smartphone apps, mobile health, mHealth, educational technology, health education, mobile phone

## Abstract

**Background:**

Pressure ulcers are areas of skin damage resulting from sustained pressure. Informal carers play a central role in preventing pressure ulcers among older and disabled people living at home. Studies highlight the paucity of pressure ulcer training for informal carers and suggest that pressure ulcer risk is linked to high levels of carer burden.

**Objective:**

This pilot study evaluated a smartphone app with a specific focus on pressure ulcer prevention education for informal carers. The app was developed based on the principles of microlearning. The study aimed to explore carer perspectives on the acceptability of the app and determine whether the app increased knowledge and confidence in their caring role.

**Methods:**

In this concurrent mixed methods study, participants completed quantitative questionnaires at baseline and at the end of weeks 2 and 6, which examined caregiving self-efficacy, preparedness for caregiving, caregiver strain, pressure ulcer knowledge, and app acceptability and usability. A subsample of participants participated in a “think aloud” interview in week 1 and semistructured interviews at the end of weeks 2 and 6.

**Results:**

Of the 32 participants, 23 (72%) participants completed the week 2 and 16 (50%) completed the week 6 questionnaires; 66% (21/32) of carers participated in qualitative “think aloud” interviews, and 18 (56%) also participated in semistructured interviews at week 2, and 13 (41%) at week 6. Pressure ulcer knowledge scores significantly changed (*F*_1,6.112_=21.624; *P=*.001) from baseline (mean 37.5; SE 2.926) to the second follow-up (mean 59.72, SE 3.985). Regarding the qualitative data, the theme “I’m more careful now and would react to signs of redness” captured participants’ reflections on the new knowledge they had acquired, the changes they had made to their caring routines, their increased vigilance for signs of skin damage, and their intentions toward the app going forward. There were no significant results pertaining to improved preparedness for caregiving or caregiving self-efficacy or related to the Caregiver Strain Index. Participants reported above average usability scores on a scale of 0 to 100 (mean 69.94, SD 18.108). The app functionality and information quality were also rated relatively high on a scale of 0 to 5 (mean 3.84, SD 0.704 and mean 4.13, SD 0.452, respectively). Overall, 2 themes pertaining to acceptability and usability were identified: “When you’re not used to these things, they take time to get the hang of” and “It’s not a fun app but it is informative.” All participants (n=32, 100%) liked the microlearning approach.

**Conclusions:**

The iCare app offers a promising way to improve informal carers’ pressure ulcer knowledge. However, to better support carers, the findings may reflect the need for future iterations of the app to use more interactive elements and the introduction of gamification and customization based on user preferences.

## Introduction

### Background

Pressure ulcers are defined as localized damage to the skin or underlying tissue, typically over a bony prominence, resulting from sustained pressure, which may present as intact skin or open ulcer [1]. They are usually caused by prolonged sitting or lying in one position. Populations at high risk include people who are frail and old [2] and people with spinal cord injuries [3]. According to a cross-sectional study, the prevalence of pressure ulcers is between 0.40 and 0.77 per 1000 community-dwelling adults in England [4].

Pressure ulcers are a substantial source of burden. They cause pain, exudate, and odor [5], and affect a person’s ability to participate in rehabilitation [6]. Pressure ulcers are also slow to heal. Most are treated using dressings and topical agents; however, some require surgical repair. Complications include soft-tissue and bone infections. Infection can cause sepsis and even death. Annually, the United Kingdom National Health Service (NHS) treats 202,000 people for pressure ulcers, at a yearly cost of £571.98 million (US $660.23 million) [7].

The essential elements of pressure ulcer prevention and management are the following: providing appropriate support surfaces (eg, pressure relieving cushions and mattresses), conducting regular skin inspections, supporting patients to keep moving, ensuring that incontinence and moisture are managed, and maintaining adequate nutrition and hydration [8]. Regarding nursing management, depending on ulcer severity, most people with pressure ulcers receive between 1 and 3 nursing visits per week for wound care [9].

Studies exploring the factors influencing the implementation of evidence-based practice in pressure ulcer prevention and management in community settings have identified how health care practitioners regard informal carers as central to both pressure ulcer prevention and wound healing [10]. In total, 4 recent studies have explored carer input in pressure ulcer prevention and management and their perspectives of the factors affecting pressure ulcer care in home settings [11-14]. The findings emphasized high levels of carer burden and highlighted the paucity of carer training and the importance of communication with health care practitioners.

### Supporting Carers

In this study, the term “informal carer” is defined as someone providing unpaid care to an older dependent person where there is an existing social relationship (eg, a spouse or other relative). All subsequent references to carers will be to those working in this informal capacity. In 2015, NHS England pledged to raise the profile of such carers and how to support them [15]. Recent systematic reviews suggest that web-based interventions may result in a range of improved health outcomes for carers, including reductions in depression, stress, anxiety, social isolation, and relationship problems [16,17]. Moreover, these studies have suggested that robust web-mediated carer education has the potential to enhance management of the caring role with a concomitant reduction in the requirement for health care practitioner input.

With improved accessibility of smartphone devices, the role of smartphone health care apps is expanding. Smartphone apps can support carers by providing access to information, support, and resources at any time where the person has internet connection. App information is also easier to update and alert users to (via a notification in the app) than equivalent paper versions, which will be more expensive and difficult to ensure that users are reached. Previous studies have assessed the use of health care apps among carers of people with cancer [18] and carers of people living with dementia [19]. Although these studies found a positive attitude toward apps among carers [18,19], they also identified barriers to their use including concerns about time constraints and not being familiar with technology [19].

### Contribution of This Study

To the best of our knowledge, no health care app has been evaluated among carers of people at risk of pressure ulceration. The aim of our study was to (1) explore carer perspectives on the acceptability and usability of a pressure ulcer app and (2) determine whether the app increased carers’ knowledge and confidence in their caring role.

## Methods

### Design

This was a 6-week, concurrent mixed methods pilot study in which participants were given access to a smartphone education app which had specific focus on carers and the care, management, and prevention of pressure ulcers. The study involved two components: (1) web-based Qualtrics-based questionnaires, completed by carers in weeks 1, 2, and 6, and (2) “think aloud” interviews with carers in week 1 and semistructured interviews with carers in weeks 2 and 6. The Good Reporting of a Mixed Methods Study guidelines [20] were adhered to in the reporting of this study.

### Ethics Approval

Ethics approval for the study was granted by the School of Health Sciences Research Ethics Committee at City, University of London (ETH1819-1600), and relevant governance approvals were received from the local NHS provider organization.

### iCare App

The app was developed by Care City, a Community Interest Company, which aims to work with residents and organizations to improve health and well-being in Northeast London, by bringing together health, social, and third sector partners; technology experts; and researchers. Using the Agylia Learning Management System, the app design and format were shaped by the principles of microlearning, in which short, focused pieces of content are provided to an audience, when and where they need it [21]. The app’s content was organized into 14 units, with each unit consisting of a video presentation, written information, and interactive learning objects (Table 1). Each of the learning units was designed to take approximately 3 minutes to complete. The content of the app reflected information contained within an educational pack for carers that was developed by the local NHS provider organization [22]. The app was available to download on iPhone and Android devices.

**Table 1:** iCare app—learning unit topics and unit format.

Unit number	Learning unit topic	Learning unit format
1	What are pressure ulcers?	Video (2 minutes 46 seconds)
2	Frequently asked questions about pressure ulcers	Video (40 seconds), written frequently asked questions, and pictures of pressure ulcers
3	Five things you should know about keeping people moving	Bullet-pointed list and interactive components
4	Five things you should know about keeping skin healthy	Bullet-pointed list and components
5	Five things you should know about nutrition	Bullet-pointed list and interactive components
6	Five things you should know about support surfaces	Bullet-pointed list and interactive components
7	How to ensure adequate nutrition?	Video (1 minute 19 seconds) and interactive components
8	How to help people keep moving?	Video (1 minute 40 seconds) and interactive components
9	How to keep skin healthy?	Video (1 minute 27 seconds) and interactive components
10	How to support people at risk effectively?	Video (1 minute 37 seconds) and interactive components
11	Pressure ulcer triggers	Interactive checklist
12	Skin inspection guide	Interactive checklist
13	Sources of help	Color-coded reference chart
14	Identifying who is at risk of getting a pressure ulcer	Color-coded reference chart

### Sampling and Recruitment

The study was conducted in London, England. Individuals meeting the following inclusion criteria were eligible to participate: (1) aged ≥18 years, (2) identifiable as an informal carer for a person with or at risk of pressure ulcer, (3) able to participate in the interview in English, and (4) have access to an iPhone or iPad or Android device.

For pilot studies, the sample size for quantitative components is suggested to be 30 [23,24], which will allow parameter estimates and loss to follow-up rates for subsequent large studies. The sample size for the “think aloud” interview and semistructured interviews were influenced by the concept of data saturation [25]. Given the topic area was clearly defined, a sample of 15 participants was expected to achieve data saturation. Previous studies using the “think aloud” approach to usability testing for health care apps have used sample sizes of 10 [26] and 24 [27], respectively.

The study was advertised on posters displayed in public areas on NHS sites in East London (including general practitioner surgery and rehabilitation wards). The study was also promoted by Care City staff attending local carer support group events. At these events, staff explained the purpose, methods, and intended uses of the study. They also explained that, depending on carer preference, participation will entail either (1) the completion of 3 web-based questionnaires or (2) the completion of 3 web-based questionnaires and participation in 1 face-to-face “think aloud” interview and 2 additional semistructured interviews. In total, 14 events were attended, at which there were approximately 150 carers; however, not all attendees met the study inclusion criteria. In total, 29 eligible carers expressed interest in participating and were provided with a participant information sheet, and consent was obtained for their contact details to be shared with both the app registration team and the research team. According to Care City, reported barriers to recruitment included the perceived relevance of pressure ulcers and digital exclusion. Regarding the former, many of those attending the carers events did not consider the person they cared for as being at risk of pressure ulceration, and therefore did not think that the app will be of benefit to them. Regarding digital exclusion, many carers reported that they did not have access to the right technology, whereas others did not feel sufficiently technologically confident to engage with an app.

Following agreement, the app registration team set up individual user accounts and emailed carers their account details and instructions for downloading the app. Only carers who expressed interest in being interviewed as part of the study were referred to the research team, who telephoned them to confirm their ongoing interest and arrange a convenient time and location for the first interview.

### Data Collection

All participants were asked to use the app for a period of 6 weeks. Data were collected between October 2019 and April 2020.

#### Web-Based Questionnaires

##### Overview

Participation for the whole sample comprised completion of web-based questionnaires at three time points: (1) at the start of week 1, (2) at the end of week 2, and (3) at the end of week 6. The questionnaires were administered via a web-based platform, Qualtrics. The first page of each questionnaire had a consent statement. Participants were directed to complete the questionnaire only after they read the statement and agreed to participate. Participants were prompted to complete the questionnaires via automatic emails sent at the start of week 1 and at the end of weeks 2 and 6. Anyone not completing the questionnaire within 7 days from the specified date received a telephone reminder from the app registration team. Participants received a £5 (US $5.81) e-voucher after completing each questionnaire to compensate them for their time and effort.

##### Week 1 (Baseline)

The baseline questionnaire comprised 3 main sections. In the first section, participants were asked to provide demographic information pertaining to their gender, age, ethnic background, highest level of education, relationship with the care recipient, and previous care-related training and the age and gender of the care recipient and their primary diagnosis. They were also asked whether they had previously used any health app or apps.

The second section measured existing pressure ulcer knowledge using a 20-item questionnaire, which was used to produce 2 parallel forms of 12 items each, at different time points (weeks 1 and 6). Items were generated from the educational pack developed by the local NHS provider organization for carers on how they can support family members at risk of pressure ulceration [22] and the Pressure Ulcer Knowledge Assessment Tool 2.0 questionnaire for registered nurses and nursing assistants [28]. Then, the items were clustered around four categories: (1) support surfaces, (2) nutrition and hydration, (3) keep moving, and (4) skin care and inspection.

The third section measured participants’ self-reported outcomes including confidence in dealing with caregiving situations, using the Caregiving Self-Efficacy Scale (CSES) [29]. The choice of responses ranged from 1 (not at all confident) to 5 (extremely confident). This section also measured how prepared participants were for their caregiving role, using the Preparedness for Caregiving Scale (PfCS) [30]. The choice of responses ranged from 0 (not at all prepared) to 4 (very well prepared). Finally, strain related to the caregiving role was measured using the Caregiver Strain Index (CSI) [31].

The week 1 questionnaire took approximately 30 minutes to complete.

##### Week 2 (First Follow-up)

This questionnaire comprised only 1 section, in which participants were asked to complete the System Usability Scale (SUS) [32] and the Mobile App Rating Scale (MARS) [33]. The former is a 5-point Likert scale (ranging from 1=”strongly disagree” to 5=”strongly agree”), giving a global view of subjective assessments of usability. The MARS also uses a range of Likert-type scale responses. The questionnaire took approximately 10 minutes to complete.

##### Week 6 (Second Follow-up)

At the end of week 6, participants were again asked to complete the CSES, PfCS, and CSI and answer follow-up questions on their pressure ulcer knowledge. The questionnaire took approximately 10 minutes to complete.

#### “Think Aloud” Interviews

The “think aloud” interviews were conducted with a subgroup of participants at the beginning of week 1. They were conducted face to face in a place with internet access chosen by the participant. The “think aloud” approach [34] was selected on the basis that it will provide a useful reflection on the carers’ cognitive processes and attitudes while downloading and using the app for the first time. To gain experience with the think aloud method, the interviewer (PH) conducted 2 pilot interviews—one with someone who had no previous exposure to this approach and one with someone who had extensive experience.

Written consent was obtained from participants before the interview. During the interview, the participant downloaded the iCare app from either the iPhone App Store or the Google Play Store. Then, the participants were encouraged to interact with the content while the interviewer asked them to verbalize their thought processes (eg, to voice any confusion or trouble they were having while navigating the app) and attitudes toward the content. All interviews were audio-recorded with participants’ permission. At the end of the interview, the interviewer made an appointment with the participant for their first semistructured interview (refer to the following section).

#### Semistructured Interviews

The subgroup that participated in the “think aloud” interviews were invited to participate in one-on-one semistructured interviews at the end of weeks 2 and 6. The topic guide for these interviews asked about participants’ use of the iCare app since the previous interview, their perceptions of using the app, changes they had made because of using the app, their plans for continuing to use the app, best thing about the app, how the app may be improved, and what other sources of pressure ulcer information they had accessed since the previous interview. Interviews were conducted by PH via telephone. Written consent was obtained before the interview. Interviews were digitally recorded with participant’s permission. At the end of the first semistructured interview, the interviewer made an appointment for the second interview.

### Data Analysis

#### Statistical Analysis

Quantitative data were entered into SPSS and analyzed for (1) description of the sample at baseline; (2) descriptives of the sample’s mobile app rating at the first follow-up; (3) relationships between continuous and ordinal variables, using Pearson or Spearman correlations; and (4) changes in outcomes from baseline to second follow-up, using linear mixed models analyses. Descriptive statistics (eg, means and SDs) have been produced and are presented in the Results section. Data were screened to check whether they met the assumptions of parametric statistics, and appropriate inferential statistics were conducted. The statistical analysis was performed by SH.

#### Qualitative Data Analysis

Interviews were transcribed verbatim by an independent professional transcription service. Pilot data were not included in the analysis. Data were sifted and interpreted using the framework approach to qualitative data analysis [35], which allows the analytical process to be informed by issues designated in advance and new and emergent themes [36]. In the steps of this approach, transcription is followed by familiarization, coding, analytical framework development, indexing, charting, and interpreting. Deductive coding was guided by the study’s aim and used predefined codes derived from the MARS [33], Service User Technology Acceptability Questionnaire [37], and Treatment Acceptability Framework [38]. In total, 2 members of the research team (CM and EM) independently coded a sample of the transcripts. The remaining transcripts were coded by CM, and an analytical framework was developed. After the framework was developed and data were charted into the matrix, the data were interpreted by CM. All interpretations were discussed and interrogated by other members of the research team (EM and SH).

## Results

The quantitative and qualitative results are integrated and presented in two parts to meet the aim of the study: (1) acceptability and usability of the iCare app and (2) impact of the iCare app on carers’ knowledge and confidence in their caring role.

### Sample Characteristics

In total, 32 participants were registered with the iCare app. were carers who had attended one of the aforementioned carers events, or carers who had responded to posters advertising the study in public areas on NHS sites. Table 2 shows the characteristics of participants.

The mean age of the sample was 57.9 (SD 11.15) years, with 69% (22/32) women and 31% (10/32) men. Of the 32 participants, 11 (34%) participants had an education level of degree or above. Most participants (21/32, 66%) identified as White (British or Irish). Of the remaining participants, most identified as of South Asian origin (6/32, 19%). Although all participants (32/32, 100%) spoke English, 25% (8/32) of them spoke a different language at home.

Regarding the person the participants cared for, the mean age was 71.4 (SD 23.15) years. It is noteworthy that this distribution was bimodal with a small number of young people receiving care (aged <38 years; 6/32, 19%) and a large number of older people receiving care (aged >60 years; 24/32, 75%). More than half of those receiving care were classified as women (18/32, 56%). Among the 32 participants, there were 8 (25%) carers looking after a spouse or partner, 11 (34%) looking after a parent, and 11 (34%) looking after a son or daughter. Just more than half of them (17/32, 53%) lived with the person they were caring for.

The most common condition or disability of the person receiving care was depression (11/32, 34%), followed by rheumatoid arthritis (10/32, 31%) and osteoarthritis (9/32, 28%). Importantly, many participants reported caring for individuals with multiple conditions and disabilities (22/32, 69%).

At baseline, of the 32 participants, only 5 (16%) participants reported using a health app before, and 8 (25%) reported participating in health education training in relation to caregiving (including diabetes care, parenting for autism, and moving and handling).

In total, 66% (21/32) of the carers were recruited to the subgroup participating in the “think aloud” and semistructured interview component of the study, including 67% (14/21) women and 70% (7/10) men.

**Table 2:** Characteristics of participants and care recipients.

Characteristics					Values
**Participants (N=32)**					
	**Sex, n (%)**				
		Male	10 (31)		
		Female	22 (69)		
	Age (years), mean (SD)			57.9 (11.15)	
	**Education level, n (%)**				
		No formal education	5 (16)		
		Other	2 (6)		
		CSE^a^ or GCSE^b^ or O-level or City and Guilds or NVQ^c^ levels 1-2	6 (19)		
		A-level or higher national diploma or NVQ level 3 or diploma	8 (25)		
		Degree or equivalent	6 (19)		
		Higher degree or postgraduate qualification	5 (16)		
	**Ethnicity, n (%)**				
		British or Irish	21 (66)		
		Asian or British Asian (Indian or Bangladeshi)	6 (19)		
		Black or Black British	2 (6)		
		Other	3 (9)		
	**Relationship with care recipient, n (%)**				
		Spouse or partner	8 (25)		
		Daughter or son	11 (34)		
		Parent (mother, father, mother-in-law, father-in-law, or grandparent)	11 (34)		
		Other	2 (6)		
	**Live with care recipient, n (%)**				
		Yes	17 (53)		
		No	15 (47)		
**Care recipients (N=32)**					
	**Sex, n (%)**				
		Male	12 (38)		
		Female	18 (56)		
		Other	2 (6)		
	Age (years), mean (SD)			71.4 (23.15)	
	**Condition or disability, n (%)**				
		Depression	11 (34)		
		Rheumatoid arthritis	10 (31)		
		Osteoarthritis	9 (28)		
		Respiratory conditions	8 (25)		
		Diabetes	8 (25)		
		Dementia	8 (25)		
		Learning disabilities	5 (16)		
		Gastrointestinal conditions	4 (13)		
		Cancer	4 (13)		
		Visual problems	4 (13)		
		Cardiac conditions	3 (9)		
		Multiple sclerosis	2 (6)		
		Stroke	2 (6)		
		Other	8 (25)		

^a^CSE: Certificate of Secondary Education.

^b^GCSE: General Certificate of Secondary Education.

^c^NVQ: National Vocational Qualification.

### Loss to Follow-up

All the participants (32/32, 100%) completed the week 1 (baseline) questionnaire. Of the 32 participants, 23 (72%) participants completed the week 2 (first follow-up) questionnaire and 16 (50%) completed the week 6 (second follow-up) questionnaire, with data available for 13 (41%) carers at all 3 time points. There were no significant predictors of withdrawal (carer characteristics, care recipient characteristics, pressure ulcer knowledge, or participant-reported outcome measures) from the study at the *P*<.01 level.

Comparisons of pressure ulcer knowledge, CSES, PfCS, and CSI were between the baseline and second follow-up measure. The linear mixed models analyses ensured that all available data were used for analyses across time points.

**Figure 1:**
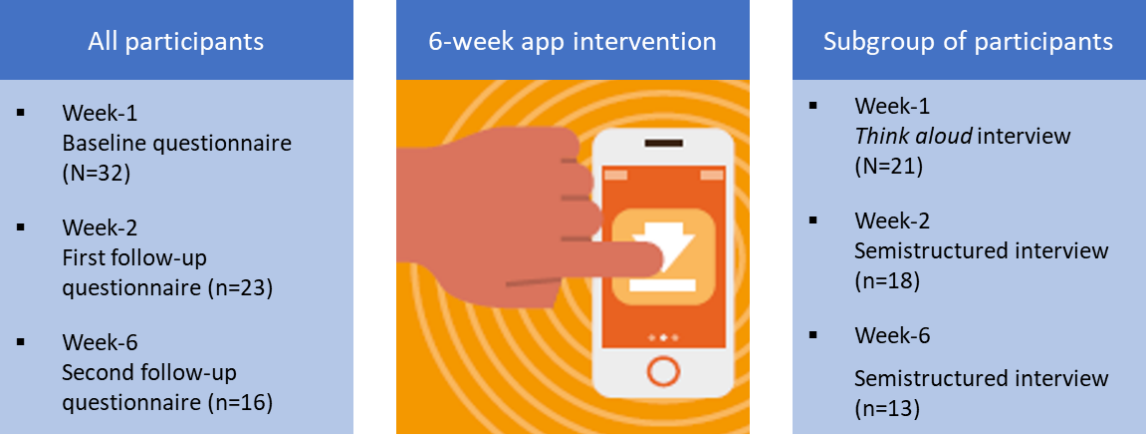
Follow-up of participants.

In terms of the qualitative subgroup, 66% (21/32) of the carers participated in the “think aloud” interview, 18 (86%) of whom went on to participate in a semistructured interview at the end of week 2 and 13 (62%) of whom also participated in the semistructured interview at the end of week 6. Figure 1 shows the follow-up of participants across the study.

### Acceptability and Usability of the iCare App

At first follow-up, participants completed SUS and MARS to provide feedback about using the iCare app. Table 3 shows the mean scale scores on these measures. SUS showed that the app has above average usability score (mean 69.94, SD 18.108). The MARS scores supported this finding and showed the app’s overall mean score (mean 3.62, SD 0.540) as being above the midpoint, with the subscales indicating that this is mainly owing to the information-conveying capabilities of the app (mean 4.13, SD 0.452) and its functionality (mean 3.84, SD 0.704). However, the engagement score (mean 3.03, SD 0.669) was midrange, and the perceived impact score (mean 2.01, SD 0.936) was relatively low based on the scale ranges.

Regarding the study’s aim, two key themes were identified from the qualitative data: (1) “When you’re not used to these things, they take time to get the hang of” and (2) “It’s not a fun app but it is informative.” The key themes, subthemes, and illustrative quotations are shown in Table 4.

The theme, “When you’re not used to these things, they take time to get the hang of,” captured participants’ perceptions and experiences relating to usability. Few participants felt proficient in the use of modern technology at baseline, with many participants describing using only the basic features of their smartphones. Varying levels of familiarity with smartphones and apps in particular were reflected in the amount of time the participants took to find and install the iCare app from the iPhone App Store or the Google Play Store, with downloading times ranging from 1 minute 22 seconds to 15 minutes 34 seconds (average 4 minutes 52 seconds).

Once the app was downloaded, some participants found navigating the content more intuitive than others. Some barriers to navigation were related to the design of the app (eg, the indistinguishable nature of pictures on the direct links [tiles] to set modules in the app), whereas others related to relatively common computing functions such as vertical and horizontal scrolling and screen orientation settings. These functions were not considered to be simple or obvious by those participants who were new to smartphone apps and were identified as a source of frustration in the “think aloud” interviews. Despite these frustrations, many participants thought that they would, with time, learn how to use the app. At the end of the second week, most participants recounted that they had become proficient in the use of the app, which reflects the high usability scores reported using the SUS in the first follow-up questionnaire. However, there were exceptions, including 2 participants who had forgotten their passwords and who had been unable to reset them again. An area that remained as a concern across the 6 weeks was the size of the font on the app. Therefore, some participants expressed a preference for printed forms of information or suggested that a desktop version of the app be made available. However, others recognized that having pressure ulcer information in an app on their mobile devices ensured that advice and support were always available.

The second theme, “It’s not fun but it is informative,” captured participants’ perceptions of and experiences with the performance of the app in terms of conveying information and its functionality and engagement. Participants who were familiar with digital technologies highlighted a missed opportunity by creators to generate an experience beyond the content itself and drew attention to the advantages of app-to-app linking and game mechanisms, which were missing from the iCare app. These participants felt that the addition of these features would have increased their engagement with the app. In contrast, some participants were irritated by animated features (such as the use of flip cards) because they required more user effort. Although participants disagreed about whether the app should be more entertaining, several participants agreed that great customization and more personalized content would have increased engagement. Regarding customization, participants suggested the addition of bookmarking and favoriting tags, which would have allowed them to return quickly to preferred content, and an activity tracker, which would have tracked their progress. Regarding personalization, a participant suggested the addition of an algorithm that will generate content that is relevant to each user’s personal circumstances. These findings may explain the reasons for the midrange scores for engagement on MARS in the first follow-up questionnaire.

Despite these limitations, participants were united in their description of the app as one that was informative. The highlight for many were the videos, in which the presenter was commended for her pace and use of plain English and for providing a welcome break from the written content. Regardless of baseline levels of knowledge, all participants (32/32, 100%) liked the microlearning approach and endorsed it across the 6-week period. These findings contextualize the high information scores on MARS in the first follow-up interview. Participants described having to juggle their caring responsibilities alongside other responsibilities and that the time they had available to dedicate to learning about pressure ulcers was limited.

**Table 3:** App-related scale scores at the first follow-up.

Scales and parameters		Scores, mean (SD)
System Usability Scale (score range 0-100; higher scores indicate greater usability)		69.94 (18.108)
**Mobile App Rating Scale (score range 1-5; higher scores indicate better rating)**		
	Engagement	3.03 (0.669)
	Functionality	3.84 (0.704)
	Esthetics	3.46 (0.876)
	Information	4.13 (0.452)
	Subjective quality score	3.17 (0.978)
	Perceived impact of the app	2.01 (0.936)
	Quality of the app	3.62 (0.540)

**Table 4:** Themes, subthemes, and illustrative quotes (acceptability and usability of the iCare app).

Themes and subthemes		Quotes
**1. When you are not used to these things, they take time to get the hang of**		
	Limited proficiency in the use of modern technology	“I don’t use mobile phones very often, only for rings.” [think aloud; P104] “I don’t really do apps...I just use the phone to check my, have I got a text message.” [think aloud; P108] “I don’t often use [the phone] as a web, for webbing.” [think aloud; P114] “I’m not very digitally minded...literally, I brought my iPhone to take pictures.” [think aloud; P105]
	Learning over time	“I went to school in the 70s and university in the 80s, so this is not my kind of thing but I could adopt it, I could try.” [think aloud; P116] “The more I use it, I’ll get the hand of it better.” [think aloud; P101] “Many people love technology, I love it, I’m crap at it but I want to try and learn.” [think aloud; P110] “I find it okay because I’ve got used to it now. I’ve looked at it a few times and then I get used to it.” [week 2; P114] “It seems pretty easy to navigate once you know how it works...Once you learn how to use it, it’s pretty intuitive.” [week 6; P117]
	Small font size	“I have to wear glasses to read and I get tired eyes, watery. I think the writing is, should be a bit bolder.” [think aloud; P108] “I think I have already said it before, I think the wording needs to be a bit bigger and bolder.” [week 2; P109]
**2. It’s not fun, but it is informative**		
	Creating an experience beyond the content itself	“I think perhaps you could consider other things like linking it to other systems. For example, like Patient Access...It’s like a GP [general practitioner] practice app where patients can log in, book appointments, repeat prescriptions and things like that. Perhaps you could link it to that because on there, there’s information and support for carers as well.” [think aloud; P106] “There’s nothing about connecting with...other carers. Nothing about having a discussion about something that you’ve just seen...You could gamify this, that would be more fun...because we spend the entire time reading which is, I get fed up with...being told ‘read this’...I don’t have the time, the energy or the capacity.” [think aloud; P121] “I suppose they are trying to make it a little bit more interesting, but they could have just done it as bullet points.” [think aloud; P104] “Because it is not a game. I don’t see [the point] of an extra click. And it makes me feel like I am doing an exam, a multiple-choice exam and it doesn’t make me feel like this is something [I’m going to want to do], I think I would get bored of it.” [think aloud; P111]
	Customization and more personalized content	“I think it would be useful to have some favorites, so sections that you know you’d want to go back to more easily.” [think aloud; P106] “There isn’t any [customization]. It’s led by the app. It’s just a whole bunch of lines. I can’t customize anything... There isn’t anything that says, I’ve done this bit, and these are the bits that are next.” [think aloud; P121] “I don’t need that [information] at the moment but if it [was] relevant to my situation... What do they call them now... flow chart! Now that would be useful...so you’re going down a tree until you hit the specific point that you are looking for...I think you have to try and tailor these things.” [think aloud; P113]
	Good use of videos	“I quite like the video content...You don’t want to just read loads and loads and loads of information.” [think aloud; P106] “I like the way she is talking...a good pace and she was very clear in describing what to look out for...the language she used – it wasn’t really hard terminology.” [think aloud; P107]
	Information is short and to the point	“Everything seems just short and to the point to keep me engaged because, especially as the care you just, your concentration level is just, you’ve just got to be on it, you’re doing other things and also tired...I just need something to spark a little something in me and be simple.” [think aloud; P121] “It’s not really a fun topic, but it’s very interesting...It was just concise information that someone in my position would need to know, it wasn’t [over the top] with lots of unnecessary information. It was just enough so that I know what to look for and what to do.” [week 2; P118] “I think it’s very quick and straight to the point most of the time...it’s a very good introduction.” [week 6; P119]

### Impact of the iCare App on Carers’ Knowledge and Confidence in Their Caring Role

Mean scores for pressure ulcer knowledge and participant-reported outcome measures (ie, CSES, PfCS, and CSI) at baseline and 6 week follow-up are reported in Table 5. Regarding the Pressure Ulcer Knowledge Assessment, at baseline, participants had a relatively low score on a scale of 0 to 100 (mean 37.5, SD 16.55). Items on the questionnaire were clustered around four key themes: (1) support surfaces, (2) nutrition and hydration, (3) keep moving, and (4) skin care and inspection. Knowledge—albeit limited—was mainly based on nutrition and hydration (mean 46.8, SD 26.7) and keep moving (mean 37.50, SD 25.40) scale scores. There were deficits in skin care and inspection knowledge (mean 25.63, SD 27.47) and support surfaces knowledge (mean 33.04, SD 20.6).

Regarding participant-reported outcome measures, at baseline, PfCS scores (mean 19.59, SD 6.339) indicated that the group was between “somewhat” and “pretty” well prepared for caring, and CSES scores showed that they were, on average, “somewhat” confident in their ability to care (mean 3.10, SD 0.815). Regarding CSI, carers scored a mean of 11.91 (SD 6.428), representing strain “sometimes.”

Overall, pressure ulcer knowledge scores significantly changed (*F*_1,6.112_=21.624; *P*<.001) from baseline (mean 37.5, SE 2.926) to the second follow-up (mean 59.72, SE 3.985). From the subscale scores, this difference was likely owing to changes in the “support surfaces” knowledge category, which increased from baseline scores of a relatively low mean of 33.04 (SE 3.653) to a relatively high score (mean 71.11, SE 3.906). Trends toward significant increases in knowledge were found from the subscales for “nutrition and hydration,” “keep moving,” and “skin care and inspection.”

There were no significant results pertaining to participant-reported outcomes across the 6-week period; the PfCS (baseline: mean 27.59, SE 1.121; second follow-up: mean 28.11, SE 1.110; *P*=.60), CSES (baseline: mean 3.10, SE 0.144; second follow-up: mean 3.38, SE 0.172; *P*=.12), and CSI (baseline: mean 11.91, SE 1.136; second follow-up: mean 12.85, SE 1.425; *P*=.47) did not show any significant change.

Regarding this study’s aim, only 1 key theme was identified from the qualitative data: “I’m more careful now and would react to signs of redness.” This theme describes participants’ reflections on the new knowledge they had acquired, the changes they had made to their caring routines as a result of this new knowledge, their increased vigilance for signs of skin damage, and their intentions regarding the app going forward. Related subthemes and illustrative quotations are shown in Textbox 1.

Participants reported acquiring new knowledge as they progressed through the different modules. Before using the app, some participants had only a rudimentary understanding of the factors contributing to pressure ulcer development, as shown in the baseline pressure ulcer knowledge scores, and had not considered the person they cared for to be especially vulnerable to pressure ulceration because they were neither wheelchair users nor confined to bed. At follow-up, several participants described how they had changed their caring routines as a result of this new knowledge, particularly routines related to movement, patient positioning, and moving and handling. However, most participants felt that the person they cared for was not at high risk of pressure ulceration. As such, the primary learning outcome had not been a change in caring behavior, but an improved understanding of the dangers of pressure ulcers and an increased readiness to react to signs of skin damage. Given that most participants felt that the person they cared for was not at high risk of pressure ulceration, at the final follow-up, most of them felt that they had learned enough about the prevention and management of pressure ulcers and did not envisage returning to the app in the immediate future. However, approximately all participants wanted to retain the app in case their circumstances were to change.

**Table 5:** Pressure ulcer knowledge and participant-reported outcome measures.

Tools and parameters		Baseline, mean (SE)			Second follow-up (week 6), mean (SE)			Test of fixed effects of time		
							*F* (*df*)		*P* value	
**Pressure Ulcer Knowledge Assessment (score range 0-100)**										
	Support surfaces	33.04 (3.653)			71.11 (3.906)			50.415 (1,22.457)		<.001
	Nutrition and hydration	46.88 (4.538)			58.62 (4.302)			6.122 (1,21.624)		.02
	Keep moving	37.50 (4.490)			57.58 (6.857)			7.365 (1,16.949)		.02
	Skin care and inspection	25.63 (4.856)			46.90 (7.611)			7.349 (1,17.299)		.02
	Total score	37.25 (2.926)			59.72 (3.985)			29.452 (1,17.850)		<.001
Preparedness for Caregiving Scale (total; score range 0-32)		27.59 (1.121)			28.11 (1.110)			0.286 (1,16.640)		.60
Caregiving Self-Efficacy Scale (mean item; score range 1-5)		3.10 (0.144)			3.38 (0.172)			2.716 (1,16.617)		.12
Caregiver Strain Index (total; score range 0-24)		11.91 (1.136)			12.85 (1.425)			0.553 (1,14.602)		.47

**Theme, subthemes, and illustrative quotes (impact of the iCare app on carers’ knowledge and confidence in their caring role).**

**Theme**

- I’m more careful now and would react to signs of redness

**Subtheme 1**

- Acquiring new knowledge
- Quotes
  “There are two pictures and I can see with the pictures it’s going to the lady’s heel, which shows obviously that if you’re sitting too long with your feet up on a surface you could develop a pressure ulcer on your heel, which is something I wouldn’t have ever thought of...So, I think there is a lot of good information there, already I can see things that I never knew about pressure ulcers, I just thought it was for people in bed.” [think aloud; P118]
  “That was really, really interesting...You mainly assume, I know about people in wheelchairs, people that are bed ridden but I didn’t realize that it could also be people like my mum that’s not well for a couple of days...So that’s really interesting...that’s shocking.” [think aloud; P104]

**Subtheme 2**

- Changing care routines
- Quotes
  “I don’t pull him up the bed anymore...I’m turning him more...” [week 2; P101]
  “I’ve just made sure, I guess that when I put my wife on the bed...I should lift her up and not drag her...I’m [also] looking...I keep an eye on [her skin]. And one district nurse, some time ago, gave me a [skin barrier] spray [to protect it from excessive moisture], which I spray. But I guess as a result of this video, I’m spraying it more often.” [week 2; P109]

**Subtheme 3**

- Alert to signs of redness
- Quotes
  “Well I certainly know the signs of redness now...so obviously there’s a sort of thing to look out for.” [week 2; P110]
  “If my mother did have bedsores...I’d know what to look for...Whereas before I had the app, I wouldn’t have had a clue really.” [week 2; P112]
  “I understand when I’m looking at something now better about soreness.” [week 2; P114]
  “The little red, I wouldn’t have ever thought of that, if I’d seen a red mark, I would have just though, oh, wouldn’t have thought much of it. But after looking at this, if I ever saw anything like that then that would prompt me to see further help.” [week 2; P118]

**Subtheme 4**

- Learned enough for now and keeping the app in case of changing circumstances
- Quotes
  “There’s only so much of it that’s relevant to me at the moment. But I know if I need to, like if my godfather for instance gets worse...then I will be able to refer back to it. Yeah, so...if the situation comes up then it’s good to have it...I’ll definitely keep it on my phone.” [week 6; P108]
  “I don’t [use it] as my wife hasn’t, at the moment anyway, hasn’t got the starting of an ulcer... [But] I’d like to keep it there...I will refer to it from time to time, because it’s always a good idea to keep on top of the situation.” [week 6; P109]
  “Because I know it’s there if I need it, but like I say, the person I look after, they’re quite mobile and I’m quite aware to look out for things...I know its there so I can go back to it...It’s only like if the situation occurred, I might go and double check something.” [week 6; P119]

### Withdrawal Analysis

There were no significant baseline predictors of withdrawal from the study at the *P*<.01 level. Only pressure ulcer knowledge regarding mobility was associated with withdrawal from the study at the *P*<.05 level, with an odds ratio of 1.035 (95% CI 1-1.071; *P*=.047). A 10-point increase in this knowledge increasing the chances of withdrawal by 3.5% (original probability of 16/32, 50%, with related odds of 1).

## Discussion

### Principal Findings

Pressure ulcers are a significant source of burden to informal carers [11-14]. Smartphone apps offer a promising way to support carers by providing access to information and resources at any time where the person has internet connection [18,19]. To the best of our knowledge, no health care app has been evaluated among carers of people at risk of pressure ulceration. The aim of our study was to explore carer perspectives on the acceptability and usability of a pressure ulcer app and determine whether the app increased carers’ knowledge and confidence in their caring role.

Despite wide variability in the ease with which carers were able to download and access the app on first use, we found relatively high levels of usability and acceptability among our sample, which comprised informal carers with and those without previous exposure to health care apps. We did not use SUS and MARS at the second follow-up to limit responder burden (especially as loss to follow-up was a concern), but taking these measures again (in a full trial) will be helpful to determine usability or acceptability following a long period with the app, as users mentioned that it will take time to become familiar with the app in the interviews. The information quality was deemed to be especially useful, and participants demonstrated that it improved their knowledge related to pressure ulcer prevention over the pilot period. Although retention of knowledge over the long term is hard to predict, several participants expressed their intention to retain the app and return to the content if they needed to in the future.

The microlearning approach was positively received by participants, who enjoyed the short focused pieces of content. Participants described having to juggle their caring responsibilities alongside other responsibilities and that the time they had available to dedicate to learning about pressure ulcers was limited. iCare does not represent the first use of the microlearning approach in health care apps for informal carers; for example, it is an approach adopted in a mobile app for supporting dementia relatives [39]. However, this study is potentially the first to report carer perspectives of and experiences with knowledge acquisition and skill development using this approach.

There were no significant results pertaining to changes in participant-reported outcomes across the 6-week period. In its current form, the app is generally didactic and underdeveloped in terms of customization and personalized content, which can include, for example, reminders for tasks and deadlines and live support for carers via groups and chats with other carers and professionals. The inclusion of such features can potentially address outcomes including preparedness for caregiving, caregiver self-efficacy, and caregiver strain. This is supported by Grossman et al [40] who suggested that the integration and interaction of five types of app functions successfully relieved caregiver burden: (1) information and resources; (2) practical problem-solving involving behavioral solutions, medication management, safety, and personal health record tracking; (3) memory aids; (4) family communication, including coordinating care; calendars for appointments and sharing; medical and emergency contact lists; and ability to share important information, photos, and messages among caregivers and family members; and (5) caregiver support (ie, care for the caregiver).

### Limitations

We met the sample size for a small pilot study to provide some indicative parameters that can be built upon for a large randomized controlled trial. However, most participants felt that the person they cared for was not at high risk of pressure ulcers. It would have been useful to have had the pressure ulcer risk assessment score (eg, scores generated using validated tools such as the Waterlow [41] and Braden [42] scales) for the person receiving care to better understand the context within which informal carers were providing care. Furthermore, the inclusion of a larger proportion of carers of people at higher risk of pressure ulcers may have produced more promising results.

The use of linear mixed models analyses helped with the loss to follow-up by allowing all available data to be used; however, the reasons for dropout need to be investigated further.

### Recommendations

This study has demonstrated that microlearning (presenting bite sized chunks of information) is acceptable and useful for users; thus, it is a strategy that can be pursued in further studies and apps of this nature, especially with carer users. However, this must be done with careful thought to the accessibility of mobile apps among the wider population. Not having either a suitable device or an internet connection is the first barrier to the usefulness of mobile apps and may exacerbate inequalities and care. Solutions may include provision of devices and training on downloading and using apps for those first-time users who require additional support, the costs of which will need to be taken into account in any wide-scale roll out. There are also some limitations in the current implementation of the iCare app, which, if addressed, can improve its usability and usefulness. Increasing interactive elements, gamification (potentially using evidence-based behavior change techniques), and customization based on user preferences are potential alterations that can achieve better results on carer-based outcomes.

### Conclusions

This study provides insight into the perceptions of carers on the acceptability of the iCare app and the impact of the app on their pressure ulcer knowledge and confidence in their caring role. The mixed methods analysis found that the app was acceptable to most participants, who endorsed the microlearning approach and perceived the app to be highly informative. In addition, at the end of 6 weeks, carers demonstrated a significant increase in their pressure ulcer knowledge. However, there were no significant results pertaining to participant-reported outcomes. The findings may reflect the need for upcoming iterations of the iCare app to use more interactive elements and introduction of gamification and customization based on user preferences. Future studies will need to capture the risk assessment scores for the person receiving care and sample a broad range of informal carers, including carers of people at high risk of pressure ulceration.

## Abbreviations

CSES: Caregiving Self-Efficacy Scale
CSI: Caregiver Strain Index
MARS: Mobile App Rating Scale
NHS: National Health Service
PfCS: Preparedness for Caregiving Scale
SUS: System Usability Scale
